# Psychometric properties of the Persian version of the Engaged teachers Scale (ETS)

**DOI:** 10.1186/s12909-024-05584-y

**Published:** 2024-05-24

**Authors:** Maryam Akbarilakeh, Soleiman Ahmady, Faezeh Shademani Farhang, Azizollah Arbabisarjou, Mohamed Hassan Taha

**Affiliations:** 1 Educational Development Center, Guilan University of Medical Sciences, Rasht, Iran; 2 Department of Medical Education, School of Medical Education and Learning Technologies, Shahid Beheshti University of Medical Sciences, Tehran, Iran; 3 School of Medical Education and Learning Technologies, Shahid Beheshti University of Medical Sciences, Tehran, Iran; 4 Department of Nursing, School of Nursing and Midwifery, community nursing research center, Zahedan University of Medical Sciences, Zahedan, Iran; 5 College of Medicine and Medical Education Centre, University of Sharjah, Sharjah, United Arab Emirates

**Keywords:** Psychometric properties, Cognitive engagement, Emotional engagement, Social engagement

## Abstract

**Background & Aim:**

Teacher engagement can be defined as the teachers’ effort and attention to teaching professional tasks, adequate emotions and commitment to relationships with students and colleagues in classroom and school. The Engaged Teacher Scale (ETS) is the frequently used scale, measuring teacher engagement developed by Klassen et al., 2013 in the UK, and consists of four dimensions: cognitive engagement (CE), emotional engagement (EE), social engagement with students (SES), and social engagement with colleagues (SEC). Therefore, the aim of this study was to measure the psychometric properties of the Persian version of the Engaged Teachers Scale (ETS).

**Materials & methods:**

A total of 123 teachers who worked at Shahid Beheshti University of Medical Sciences, Tehran, Iran, were included in this study. The study participants were selected using the convenience sampling. The Persian version of a 16-item scale developed by Klassen et al. was validated by this study. Face and Content validity index and reliability were assessed after translation and cultural adaptation, and also construct validity was calculated by confirmatory factor analysis using the PLS22.

**Results:**

Of the 123 study participants, 74 (60.01%) were females and 49 (39.9%) were males. The mean age of the subjects was about 30–40 years. The majority of the study participants were general practitioners (*n* = 75; 60.9%) and others were from different specialists. Content validity among 15 experts was 0.88. Confirmatory factor analysis for all 16 items loaded across four factors, and this four-factor scale showed a good fit in the Iranian community. Reliability using Cronbach’s alpha was 0.79. The value of root mean square error of approximation (RMSEA) was 0.0094 with the 99% confidence interval, and also the goodness of fit index GFI value was 0.98.

**Conclusion:**

The Persian version of ETS had good validity and reliability in Iran and could be a useful tool for measuring the teacher engagement factors of faculty members that can be used by teachers and educational administrators.

**Supplementary Information:**

The online version contains supplementary material available at 10.1186/s12909-024-05584-y.

## Introduction

All over the world in the higher education system, teachers play a crucial role in the excellence of education by articulation between the school, the students, and society. They also have a key role in the teaching-learning process by transferring their knowledge and experiences to the students. Also, teachers play a fundamental role in the students’ personal and professional development. Previous studies showed that teacher work engagement being a phenomenon was associated with the teacher’s performance [1].

Engaged teachers are more likely to take active roles in their work and make contributions to school life. Teacher engagement is the state of mind merged in the teaching process. Engaged teachers are spending their energy, time and attention on teaching, and have positive emotional response, commitment in suitable relationships with students and colleagues [2].

They also benefit from school environments, via increasing the autonomy, and having good relationships with their colleagues who have greater opportunities for professional development. Therefore, it is important to understand the vital role of teacher work engagement in education. Effective education is very dependent on teachers who are cognitively, emotionally and socially engaged in their work. Research shows that teacher work engagement is directly transferred to students. Because teacher engagement can play an important role in the teaching-learning process in the higher education, being aware of its status and planning to improve it can have positive effects on both quality education and teacher development. Accordingly, we tried to find a tool that measures this feature [3].

We found that one of the frequently used tools was developed by Klassen et al. (2013) in the UK developed Engaged Teachers Scale (ETS), measuring teacher engagement, and consists of four dimensions: cognitive engagement (CE), emotional engagement (EE), social engagement with students (SES), and social engagement with colleagues (SEC), indicating good psychometric characteristics in different samples. Also, the final version of the scale was applied to a sample of 321 teachers and the confirmatory factor analysis results evidenced that the instrument showed good fit indices (X2/gl = 292.67 (98); CFI = 0.97; GFI = 0.90; NFI = 0.96; RMSEA = 0.08), in addition to Cronbach’s alpha coefficients, ranging from 0.79 to 0.84. Moderate to strong positive correlations were also found between the teachers’ engagement using the ETS and their work engagement, as well as their self-efficacy. These results are promising and recommend the use of the ETS in future research involving this professional group [4]. This justifies the construction of scale, and permits understanding and assessing work engagement of teachers, which is fundamental to understanding the psychological processes underlying high-quality teaching [5].

In medical education in Iran, no studies were conducted on the scale; however, less research is performed on assessing teachers’ engagement work [6]. Given the importance of teachers’ engagement work; therefore, more studies are needed to explore teacher engagement using a valid and reliable tool. Accordingly, this study aimed to measure the psychometric properties of the Persian version of the Engaged Teachers Scale (ETS) in Iran.

## Materials & methods

### The study participants and setting

A total of 123 teachers, who worked at different teaching levels at Shahid Beheshti University of Medical Sciences, Tehran, Iran, were included in this study. The study participants were selected using the convenience sampling. The study inclusion criteria were as follows: being working, having at least one year of experience, and the full-time teachers. Of the 123 participants, 74 (60.01%) were females, and 49 (39.9%) were males. The age range of participants who taught in the two major teaching hospitals was between 30 and 40. The majority of study participants were general practitioners (no-=75; 60.9%) and others were from different specialists. The participants were involved in a cross-sectional research design. To survey teachers’ self-assessment, an analytical approach was used to validate the scale. Also, the study participants completed the 16-item ETS consisting of four dimensions: cognitive engagement (CE), emotional engagement (EE), social engagement with students (SES), and social engagement with colleagues (SEC), and answered on a 7-point Likert scale (0 = Never, 6 = Always). All the scales were distributed among the participants and data were collected during the COVID-19 pandemic.

### Tools

#### Engaged teacher scale (ETS)

The teacher work engagement was assessed using the ETS developed and validated by Klassen et al., (2013) consisting of 16 items and rated on seven-point Likert scales ranging from 0 (never) to 6 (always) and consisted of four dimensions: cognitive engagement (CE), emotional engagement (EE), social engagement with students (SES), and social engagement with colleagues (SEC) [1]. The scale reflects medical educators’ cognitive, emotional and social engagement that they can provide during teaching process, even when faced with their colleagues and students in an educational environment [4].

ETS shows good validity and reliability in many studies [1]. It is a self-rating instrument in which teachers indicate the extent of statements. The original version of ETS showed good fit indices, and the appropriate internal consistency indices (between 0.79 and 0.84). Based on the guidelines developed by Brislin (1970) [7] and Jones et al. (2001) [8] for the translation and adaption of research instruments, the ETS was translated into Persian.

To translate and adapt the scale to Persian, we emailed the scale developers to obtain third-party rights permission to use it, and also two professional educational psychologists who mastered the English language translated the scale from English to Persian. Also, another professional checked the semantics between the first and second translations and reached a final consensus on the initial Persian version of the scale. In addition, agreement was reached on most translated items with some discussion necessary for consensus by a panel of experts consisting of four faculty members from medicine and medical education departments. They reviewed the translated items and compared them with the original ones to maintain the translation quality. This was also translated back by two independent, bilingual experts. Also, two bilingual and independent experts provided the back translation. After comparing the two versions with one another and discussing any differences with two experts who were proficient in English, they agreed on the final version.

As a result, the Persian version of the ETS was considered appropriate for implementing by teachers for doing M.Sc. of medical education thesis completion in a medical school of Iran. After translation and cultural adaptation, face and content validity and reliability were checked, and also construct validity was calculated by confirmatory factor analysis using factor analysis software. 16 items of the ETS were as follows:

1. At school, I connect well with my colleagues.
2. I am excited about teaching.
3. In class, I show warmth to my students.
4. I try my hardest to perform well while teaching.
5. I feel happy while teaching.
6. In class, I am aware of my students’ feelings.
7. At school, I am committed to helping my colleagues.
8. While teaching, I really ―throw” myself into my work.
9. At school, I value the relationships I build with my colleagues.
10. I love teaching.
11. While teaching I pay a lot of attention to my work.
12. At school, I care about the problems of my colleagues.
13. I find teaching fun.
14. In class, I care about the problems of my students.
15. While teaching, I work with intensity.
16. In class, I am empathetic towards my students.

### Data collection Procedure

The data were collected from a printed version. Based on research of medical university, principals in Iran were contacted to request their authorization to develop the research among the teachers. With their permission, the date and time were scheduled for the data collection, when the study objectives and instructions were explained. Then, the scale forms were distributed, completed and returned at the end of the session. The participants chose to participate in the study voluntarily and completed the written informed consent form. In addition, the anonymity of any information provided was guaranteed to all respondents.

### Data analysis

Adaptation and validation steps included translation, determination of face validity, content validity, construct validity, and reliability. The Persian version of the scale was used for this study. In this study, the demographic variables, such as age and sex were evaluated.

To determine the face and content validity, the ideas of 15 medical education experts and professors of the medical education departments who worked in the field of psychometrics and teaching engagement were used. Also qualitative and quantitative methods were utilized for this purpose [9]. The Content validity index (CVI) of each item was evaluated based on simplicity, clarity, and relevance.

### The results of content validity

The mean content validity ratio was 0.85. The mean CVI was 0.91 for 15 expert members referred to Lawshe’s table, which was 0.88.The results of content validity showed that no items were omitted.

### Assessing internal consistency reliability

The reliability of the scale using Cronbach’s alpha was 0.79, indicating an appropriate level.

To measure the construct validity, confirmatory factor analysis (CFA) was performed using PLS22. The CFA for all 16 items was loaded across four factors, and this four-factor scale showed a good fit in the Iranian community.

### Ethical consideration

The study protocol was approved by the Ethics Committee of Shahid Beheshti University of Medical Sciences (ethical code = IR.SBMU.SME.REC.1399.010).

## Results

According to the value (0.921) obtained from the following equation, the model showed good fit.

$$GOF = \sqrt{0.7496\text{*}0.985} = 0.921$$

The result of the Kaiser-Meyer-Alkin (KMO) test was 0.75, showing that the sample size was sufficient. The Bartlett test also showed a significant value. The model fit indices were all acceptable according to Table 1. The fit criterion for CMIN / DF was 2.036, which is good if less than 5. In terms of comparison of items, the CFI was 0.9, which is good in the saturated model (Table 1).

**Table 1:** Indices related to the model fit and construct validity and their calculated value

Indicators	CMIN/DF	PNFI	AGFI	GFI	IFI	CFI	RMSEA
**Acceptable range**	5 − 1	> 0.5	> 0.8	> 0.9	> 0.9	> 0.9	< 0.08
**Calculated value**	2.036	0.79	0.89	0.98	0.97	1	0.0094
**Fitting status**	has it	has it	has it	has it	has it	has it	has it

**75/0 = KMO Bartlett s test of sphericity: (p-value < 0.000)**

Investigating the items related to each dimension after factor analysis showed that actor loads above 0.4 for each item based on factor analysis in the respective dimension in Table 2.

**Table 2:** Items with factor loading in four dimensions

Item number	Items	Factor CE	Factor EE	Factor SE c	Factor SE s
11	While teaching I pay a lot of attention to my work.	0.987			
8	While teaching, I really ―throw” myself into my work.	0.974			
15	While teaching, I work with intensity	0.987			
4	I try my hardest to perform well while teaching.	0.949			
10	I love teaching		0.960		
2	I am excited about teaching.		0.962		
5	I feel happy while teaching.		0.970		
13	I find teaching fun.		0.751		
9	At school, I value the relationships I build with my colleagues			0.948	
7	At school, I am committed to helping my colleagues			0.902	
12	At school, I care about the problems of my colleagues			0.947	
1	At school, I connect well with my colleagues			0.898	
14	In class, I care about the problems of my students.				0.928
16	In class, I am empathetic towards my students				0.990
6	In class, I am aware of my students’ feelings.				0.916
3	In class, I show warmth to my students.				0.899

### Examining the reliability after factor analysis

After performing factor analysis and identifying the factors, Cronbach’s alpha shows in Table 3.

**Table 3:** Reliability after factor analysis

Factors	Cronbach’s alpha
**Cognitive Engagement**	0.84
**Emotional E**	0.74
**Social E Colleagues**	0.75
**Social E Students**	086
**Total**	0.79

## Discussion

The purpose of this study was to measure the construct validity of the ETS [1] at Shahid Beheshti University of medical sciences, Iran. After performing the CFA, the model with the best fit index confirmed the four-factor model proposed by Klassen et al., (2013) who was conducted a study on the ETS, and assessed a four- factor model using the CFA. The results showed that the items of factors were significantly related to each other.

Due to the lack of validated instrument in Persian, this study was conducted to validate a Persian version of the ETS by examining its psychometric properties. For the first time, the psychometric tests of the scale were carried out, and also ETS was applied to determine the teacher engagement. Evidence emphasizes the need for a valid and reliable tool in each context surveying the teachers’ self-assessment of teacher engagement construct in higher education [9]. Collecting the data by teacher engagement measurement can inform decisions and policies in this area based on the results of accurate measurement [10].

The results of the content validity of the 16-item scale, taking into account the mean content validity of 0.88, showed that no items were omitted. The Persian version of the scale showed good content, construct validity and model fit and good reliability. In consistent with our study, the survey of teachers in Indonesia with a translated and adapted version of the scale successfully also confirmed the Klassen et al.’s model (2013) [2]. The results of this study were consistent with those found by Silva et al., 2020 reporting validity evidence for the ETS in the Brazilian context [11]. A Chinese version of the Engaged Teacher Scale (C-ETS) validated by Ho, S. K., et al. (2021) also confirmed the Kassel 2013 model, which was consistent with our study [10].

Emotional engagement of teachers is vital for doing the role and quality responsibility of a teacher. Also, other engagement factors need attention for the empowerment. Due to the strategic and social responsibility of teachers in educational environments, social engagement should be empowered [12]. This plan will improve through valid measuring teacher engagement factors. Cognitive, emotional and social engagement in teaching roles and jobs should be measured in development programs by administrators at medical universities [13]. The appropriate measurement of teacher work engagement is considered as an indicator of quality assurance, and also accreditation of education is very helpful. Well-prepared teaching engagement is valuable and contributes to teaching, learning and educational achievement [14].

### Limitations

It should be informed, however, that sample of teachers from one specific medical university in one city (Tehran) was included in this study, which is why the results found should only be generalized with caution, as the psychometric indexes presented here are restricted to this type of sample.

Also, the participants were all working in two hospitals in Tehran, and were largely from physicians and clinical settings, and thus the sample offers only limited representativeness to other populations of teachers.

In this study, the cross-country comparisons were performed. It was better to investigate the relationships between teaching engagement and other related variables for convergent validity. Larger sampling by suitable technique to ensure generalizability is also proposed for future studies. Therefore, it is recommended that future research to be investigated the association among teacher engagement, and socio-demographic and professional variables. This initial validity evidence recommends the future use of the ETS in Persian studies that aim to assess the degree of teacher engagement and its implications for these individuals’ wellbeing and quality of life in the job context.

## Conclusion

We found that ETS is appropriate for the measurement of teacher engagement factors like other previous studies. This study aimed to create a psychometrically valid Persian version of ETS in Iran. Policy makers in higher education, wishing to evaluate the validity of their policy choices in engaged teachers, could actively consider using an instrument like the ETS as a means of gaining validation evidence. Universities studies concerning teachers engagement views impact learners’ attitudes, beliefs, and emotions showed that negative beliefs impacts may be overcome learning achievements [15]. This is why we should consider more to improve teacher engagement in educational environments. This influences teachers thinking, cognition, emotions and socialization and can regulate success learning process and Learning outcomes are enhanced.

### Electronic supplementary material

Below is the link to the electronic supplementary material.

Supplementary Material 1

## Data Availability

The datasets generated and/or analyzed during the current study are not publicly available due to privacy and ethical considerations but are available from the corresponding author on reasonable request.
