# Supplementary material for: Psychometric properties of the Persian version of the Engaged teachers Scale (ETS)
Source: BMC Med Educ. 2024 May 24;24:574. doi: 10.1186/s12909-024-05584-y (PMC11127309; doi:10.1186/s12909-024-05584-y)
Supplement: Supplementary file 1 — Supplementary Material 1 [file 12909_2024_5584_MOESM1_ESM.pdf]

ENGAGED TEACHERS SCALE

Below you will find a list of statements describing your experiences as a teacher. Please indicate your personal response to each of these statements by checking the number that best represents your answer.

0 = Never 3 = Sometimes 6 = Always

	Never	Rarely	On occasion	Sometimes	Often	Frequently	Always
1. At school, I connect well with my colleagues.	⓪	①	②	③	④	⑤	⑥
2. I am excited about teaching.	⓪	①	②	③	④	⑤	⑥
3. In class, I show warmth to my students.	⓪	①	②	③	④	⑤	⑥
4. I try my hardest to perform well while teaching.	⓪	①	②	③	④	⑤	⑥
5. I feel happy while teaching.	⓪	①	②	③	④	⑤	⑥
6. In class, I am aware of my students' feelings.	⓪	①	②	③	④	⑤	⑥
7. At school, I am committed to helping my colleagues.	⓪	①	②	③	④	⑤	⑥
8. While teaching, I really "throw" myself into my work.	⓪	①	②	③	④	⑤	⑥
9. At school, I value the relationships I build with my colleagues.	⓪	①	②	③	④	⑤	⑥
10. I love teaching.	⓪	①	②	③	④	⑤	⑥
11. While teaching I pay a lot of attention to my work.	⓪	①	②	③	④	⑤	⑥
12. At school, I care about the problems of my colleagues.	⓪	①	②	③	④	⑤	⑥
13. I find teaching fun.	⓪	①	②	③	④	⑤	⑥
14. In class, I care about the problems of my students.	⓪	①	②	③	④	⑤	⑥
15. While teaching, I work with intensity.	⓪	①	②	③	④	⑤	⑥
16. In class, I am empathetic towards my students.	⓪	①	②	③	④	⑤	⑥

Cognitive Engagement = Items 4 + 8 + 11 + 15

Emotional Engagement = Items 2 + 5 + 10 + 13

Social Engagement: Students = Items 3 + 6 + 14 + 16

Social Engagement: Colleagues = Items 1 + 7 + 9 + 12

Klassen, R. M., Yerdelen, S., & Durksen, T. L. (2013). Measuring teacher engagement: The development of the Engaged Teacher Scale (ETS). *Frontline Learning Research, 2,* 33-52.
